# Cellular-V2X Communications for Platooning: Design and Evaluation

**DOI:** 10.3390/s18051527

**Published:** 2018-05-11

**Authors:** Giovanni Nardini, Antonio Virdis, Claudia Campolo, Antonella Molinaro, Giovanni Stea

**Affiliations:** 1 Dipartimento di Ingegneria dell Informazione, University of Pisa, Largo Lucio Lazzarino 1, 56122 Pisa, Italy; g.nardini@ing.unipi.it (G.N.); antonio.virdis@unipi.it (A.V.); giovanni.stea@unipi.it (G.S.); 2 Dipartimento DIIES, University Mediterranea di Reggio Calabria, Via Graziella, Loc. Feo di Vito, 89122 Reggio Calabria, Italy; claudia.campolo@unirc.it

**Keywords:** V2X, cellular V2X, VANETs, cooperative driving, 3GPP, platooning

## Abstract

Platooning is a cooperative driving application where autonomous/semi-autonomous vehicles move on the same lane in a train-like manner, keeping a small constant inter-vehicle distance, in order to reduce fuel consumption and gas emissions and to achieve safe and efficient transport. To this aim, they may exploit multiple on-board sensors (e.g., radars, LiDARs, positioning systems) and direct vehicle-to-vehicle communications to synchronize their manoeuvres. The main objective of this paper is to discuss the design choices and factors that determine the performance of a platooning application, when exploiting the emerging cellular vehicle-to-everything (C-V2X) communication technology and considering the scheduled mode, specified by 3GPP for communications over the sidelink assisted by the eNodeB. Since no resource management algorithm is currently mandated by 3GPP for this new challenging context, we focus on analyzing the feasibility and performance of the dynamic scheduling approach, with platoon members asking for radio resources on a per-packet basis. We consider two ways of implementing dynamic scheduling, currently unspecified by 3GPP: the sequential mode, that is somehow reminiscent of time division multiple access solutions based on IEEE 802.11p—till now the only investigated access technology for platooning—and the simultaneous mode with spatial frequency reuse enabled by the eNodeB. The evaluation conducted through system-level simulations provides helpful insights about the proposed configurations and C-V2X parameter settings that mainly affect the reliability and latency performance of data exchange in platoons, under different load settings. Achieved results show that the proposed simultaneous mode succeeds in reducing the latency in the update cycle in each vehicle’s controller, thus enabling future high-density platooning scenarios.

## 1. Introduction

The recent advancements in the sensing, automation, computing, communication and networking technologies for vehicles hold the promise of improved road safety and traffic efficiency and reduced fuel consumption and emissions [1]. By exploiting sensing and communication capabilities, vehicles can cooperate and extend their context awareness beyond the visual field. Cooperative vehicles will share their driving intentions with other traffic participants, thus accurately predicting what other traffic participants are going to do, and optimizing their own decisions and maneuvers accordingly [2].

Platooning is among the key cooperative driving applications. In a platoon, autonomous/semi-autonomous vehicles organize themselves in order to travel along the same lane at the same speed in a train-like manner, i.e., each one keeping a small, constant distance to the vehicle ahead. Such a driving pattern lays its foundation in adaptive cruise control (ACC) systems, which allow one to measure the distance from the preceding vehicles through automotive radars mounted on the front of the vehicle. However, ACC systems exhibit some limitations. The on-board sensors of an ACC system have a short sensing range (they can only see adjacent vehicles) and do not work reliably under adverse weather conditions [2,3,4]. Such limitations are overstepped by cooperative ACC (CACC) systems which leverage direct vehicle-to-vehicle (V2V) wireless communications. In CACC information about the maneuvering (e.g., position, speed, and acceleration, retrieved through sensor measurements) of one, i.e., the leading one, or more vehicles can be made available almost instantly to all other vehicles, in order to ensure quicker reaction to changes in traffic conditions, while avoiding platoon instability. The behavior and stability of the platoon is highly affected by the reliability and latency of messages exchanged among platoon vehicles.

So far, the IEEE 802.11 standard [5] has been mainly considered as the communication technology for platooning. In order to support platooning requirements, many literature solutions have proposed modifications to the native contention-based distributed 802.11 access protocol, which is known to suffer from unbounded delay and low reliability at high network load. Time-division multiple access (TDMA) schemes on top of 802.11 have been mainly pursued so far [3,6,7,8]. On the other hand, the Third Generation Partnership Project (3GPP) evolved towards a network-assisted device-to-device (D2D) Long-Term Evolution (LTE) communication technology [9] in Release 12. D2D communications suit particularly well localized V2V communications, since—on one hand—they exhibit lower latency than classic infrastructure-based communications, which instead traverse both an uplink and a downlink leg, and—on the other—they are still scheduled by the infrastructure, which implies that the benefits of interference management and collision avoidance are still present. Plenty of literature solutions have investigated the feasibility of D2D for vehicular communications [10,11,12,13,14,15], identifying the potentials as well as the limitations of the 3GPP-specified D2D technology to support the low latency requirements of vehicular applications in a high-speed context.

Recent efforts by industries and academia coalesced into the development of the Cellular Vehicle-to-Everything (C-V2X) technology [16], lately specifically conceived by 3GPP in Release 14 [17,18] to optimize D2D communications among vehicles under high mobility conditions. Early studies have been published mainly focusing on assessing the performance of C-V2X in supporting the delivery of cooperative awareness messages (CAMs) [19] in the one-hop neighborhood of the sender nodes [20,21] for cooperative road safety purposes. The challenges of the latter type of vehicular application are less strict than those raised by platooning, although it also relies on localized V2V communications and CAMs exchange.

Platooning is mentioned in the 3GPP Release 15 [22] among the key safety use-cases to be targeted with future evolution of C-V2X. Platooning applications exhibit unique features in terms of: (i) *topology*, since platoon members are positioned in the same lane along a chain and keep a very small inter-vehicle distance; (ii) *CAM coverage range*, which is in the order of tens of meters, instead of hundreds of meters, as in classic safety applications, since communications typically occur among adjacent platoon vehicles; and (iii) *delivery requirements* as the latency is expected to be much lower than 100 ms and the reliability greater than 90%. Such peculiarities make the analysis different from what already investigated in the literature for V2V communications and raise novel concerns which require to be addressed in a different manner. To the best of our knowledge, only very few works in the scientific literature have addressed the support of platooning applications through LTE/C-V2X and evaluated its performance quantitatively.

In particular, in [23] a resource allocation approach is proposed that controls channel and power assignment to support V2V communications in underlaying cellular network in a scenario with a chain of platoons. The evolved multimedia broadcast multicast services (eMBMS) capability of LTE is used to disseminate the messages transmitted by the leading vehicle to all vehicles in the platoon. In [24], we designed a solution according to which pooled LTE resources are allocated to the platoon by the eNodeB, which exploits the short inter-vehicle distances and the low transmission power to enable reuse of LTE resources by those nodes for which the mutual interference is weak. In that work, early simulation results were provided to showcase the timeliness of message dissemination within the platoon under ideal settings, i.e., perfect frequency reuse, while neglecting the impact of signaling procedures for getting radio resources.

In this work, we take a step forward in investigating the key design issues and choices related to the network-assisted mode specified by 3GPP for C-V2X communications over the sidelink (i.e., the PC5 reference point) [18], and focus on the challenging case of platooning. No scheduling algorithm is currently mandated by 3GPP for the eNodeB-assisted mode for C-V2X [21]. In such a context, this paper intends to provide the following main contributions:

- Overcome the current lack of either a legacy or a dominant solution to manage platooning applications with LTE/C-V2X and analyse the viability and performance of the *dynamic scheduling* [25] for V2X sidelink platoon communications. We consider two possible ways of implementing dynamic scheduling; both modes are not currently specified by 3GPP for C-V2X. With the first mode, called *sequential*, somehow reminiscent of TDMA solutions based on IEEE 802.11p—till now the only investigated access technology for platooning [6,7,8]—platoon vehicles ask the eNodeB for radio resources as soon as they receive a CAM from the preceding vehicle. The second mode, called *simultaneous*, allows all vehicles in the platoon to send their requests to the eNodeB soon after receiving the CAM from the leading vehicle, and supports *simultaneous CAM transmissions* between couples of vehicles, sufficiently spaced apart within the platoon, by using the *same resource blocks*.
- Derive theoretical bounds for the time required to disseminate CAM messages within a platoon, when varying the composition of the platoons, CAM size and network parameters, for the purpose of a preliminary comparison between the sequential and simultaneous transmission schemes.
- Carry out extensive simulations using SimuLTE [26], a system-level simulator based on OMNeT++ [27] that accurately implements the whole LTE protocol stack, from the radio propagation dynamics and access technology to the application data pattern, which we extended so as to support the CAM exchange within a platoon and the proposed scheduling solutions. Results have been provided in a multi-platoon scenario under a wide range of settings. The impact of different scheduling schemes (sequential vs. simultaneous) and resource allocation policies (based on channel quality feedbacks; with and without frequency reuse) is evaluated in a stepwise manner to better understand how each parameter affects the reliability and latency performance of the CAM exchange within a platoon and in multiple platoons.

The remainder of the paper is organized as follows. Section 2 introduces the platooning concept, whereas the main pillars of C-V2X technology are discussed in Section 3. The proposed scheduling schemes are presented in Section 4. Section 5 reports on the theoretical bounds for preliminary comparison between the sequential and the simultaneous modes. Section 6 reports details about the enhancements of the simulator and the simulation settings. Section 7 discusses the main achieved results, by describing avenues for future research, before conclusions in Section 8.

## 2. An Overview of Platooning

A platoon is a convoy of vehicles (e.g., trucks) travelling on the same lane, keeping as short headway as possible between each other. The head-of-line vehicle is called the Platoon Leader (PL), and is responsible for setting the pace for the whole platoon. The other vehicles are called Platoon Members (PMs) and they adjust their speed to keep the pace set by the PL.

A platoon is a complex system that synergically integrates sensing, communication and control technologies [28]. Each platoon vehicle contributes to the platoon stability by exchanging information with other vehicles about its current kinematics status and intended maneuvers, manipulating data from on-board sensors (e.g., cameras, LiDARs, radars installed in each vehicle), and acting on its actuators based on the received information.

**Figure 1:**
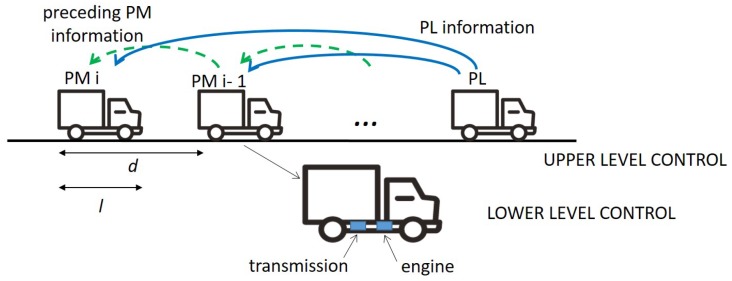
Two-level controller for the predecessor-leader control strategy.

In a typical CACC system, each vehicle includes a two-layer controller [29]. The *upper level* controller computes the desired acceleration to maintain the platoon stability, i.e., the desired safety distance from the preceding vehicle. Since the desired acceleration is not a true control input, a *lower level* controller is required to control the actuation of the vehicle in order to track the desired acceleration and so determine either a throttle (regulating the flow of the air in the burning chamber) or a brake input. We focus on the predecessor-leader control strategy, with the *upper level* controller fed by information from both the PL and the preceding PM, see Figure 1. This strategy prevents the undesirable effect of string instability due to the error amplification along the string of vehicles, which is typical of the predecessor-following control strategy, where each vehicle only communicates with the preceding one to adjust its relative position.

To derive the desired acceleration, each vehicle *i* (*i* being its position in the platoon, i≥1) relies on the position $x_{i-1}$, the speed $s_{i-1}$, and the acceleration $a_{i-1}$ of the preceding vehicle, as well as the speed $s_{0}$ and the acceleration $a_{0}$ of its PL. Precise self-position and kinematics information are available in real time on board the vehicle, whether obtained from Global Positioning System (GPS)/differential GPS or other sensing means. The distance of each vehicle to its precedent is determined by on-board sensors [7], which provide up-to-date information with negligible error. Wireless communications allow each vehicle to receive the speed and acceleration values from both the preceding vehicle and the PL. Such information is conveyed in CAM exchanged at each update cycle, regularly initiated by the PL. The cycle duration must be kept in the order of 100 ms or lower, and is set by the PL according to the speed of vehicles, the distance between them, and the size of the platoon.

Small inter-vehicle distances are typically targeted within a platoon to increase the road capacity and improve the fuel efficiency of vehicles. However, they may compromise the robustness of CACC, raising more challenges in fulfilling the reliability and latency demands of exchanged CAMs. According to [22], the traffic pattern of a platooning application entails periodic exchange of packets with a payload size in the range between 50 and 6500 bytes, and a data rate between 0.012 and 65 Mbps. Corresponding delivery requirements are the end-to-end latency (computed from the moment the CAM is transmitted by the source to the moment it is received at the destination) in the order of 10–25 ms, and the reliability between 90% and 99.999% [2,22].

Such needs motivated several literature solutions aimed at improving the delivery of platoon messages by enhancing the performance of the IEEE 802.11 protocol [3,6,7,8]. Recently, the potential of the LTE technology has been preliminarily explored to support the aforementioned platooning demands [23,24], and further benefits are expected when leveraging the C-V2X technology, whose main features are detailed in the following.

## 3. Cellular V2X: A Primer

The role of 3GPP and cellular networks in supporting vehicular communications has been steadily and rapidly growing. Granted the technological feasibility and the undisputed business opportunities for network operators, 3GPP started a feasibility study to support C-V2X communication in the first quarter of 2015. V2X is part of 3GPP LTE starting from Release 14 [17,18], with a clear roadmap for 5G networks to provide the ultra-high reliability and ultra-low latency demands of tomorrow V2X applications (e.g., autonomous driving, car platooning) [22].

There are two ways to support C-V2X communications specified in Release 14, namely over the LTE-Uu conventional cellular radio interface and over the PC5 radio interface. Communications over the LTE-Uu interface can be unicast and/or leverage eMBMS. In the latter case, a road-side unit (RSU)/eNodeB can disseminate V2X safety data towards multiple vehicular user equipments (VUEs) in a given area via eMBMS. V2V communications are enabled by the new Evolved Universal Terrestrial Radio Access (E-UTRA) capability designated as PC5, also known as *sidelink* at the physical layer, as opposed to the conventional uplink (UE-to-eNodeB) and downlink (eNodeB-to-UE). The specifications of this interface, which is the focus of this paper, have evolved since Release 12, as described in the next subsection.

### V2V Communications: From LTE D2D to C-V2X

In an LTE network, UEs are under control of one or more eNodeBs, which are responsible for allocating time-frequency resources to UEs. Scheduling of transmissions is performed in a centralized manner at the eNodeB’s Medium Access Control (MAC) layer. At every Transmission Time Interval (TTI) of 1 ms, the eNodeB allocates Resource Blocks (RBs) to backlogged UEs according to its own scheduling policy. The number of RBs allocated to one transmission depends on the Modulation and Coding Scheme (MCS) chosen by the eNodeB for that transmission. To this aim, the eNodeB can make use of a Channel Quality Indicator (CQI) reported by the UE, which is a discretized measurement of the channel quality it perceives. In the downlink, the eNodeB performs the transmission to the intended receiving UE on the allocated RBs, whereas in the uplink the UE transmits to the eNodeB as soon as it has received a transmission grant from the eNodeB including the information about the MCS and the RBs it can use.

The conventional LTE architecture requires that every communication goes through the cellular infrastructure (via the LTE-Uu interface), hence it does not natively support direct V2V communications (crucial for cooperative driving services); end-to-end latency could increase due to message passing through infrastructure nodes before being redistributed back to destination vehicles [1]. This issue can only be circumvented by resorting to device-to-device (D2D) communications [30]. Since LTE Release 12, the 3GPP has provided specifications to allow direct message delivery between terminals in proximity through D2D communications (also known as Proximity Services, ProSe) [9]. ProSe are tailored to public-safety applications and have been optimized for robust operation and long lifetime of battery-power devices, with a focus on broadcast/multicast communications in low-density scenarios.

Starting from Release 14, 3GPP focused on specifically supporting V2V communications. They exhibit peculiar data traffic patterns and challenging requirements in terms of high reliability and low latency to be met under high-speed and high-density conditions. Similarly to ProSe, V2X communication utilizes single-carrier frequency division multiple access, while supporting 10 and 20 MHz-wide channels. Each channel is divided into 1ms-long subframes, as the TTI. A group of RBs in the same subframe composes a *subchannel* that can be used to transport both signaling and data transmissions.

Two new high-level deployment configurations [17] have been designed for V2X (that extend Mode 1 and Mode 2 defined for ProSe):

- *Mode 3* (a.k.a. scheduled), according to which resource scheduling and interference management over the PC5 interface are assisted by eNodeBs via control signaling over the LTE-Uu interface.
- *Mode 4* (a.k.a. autonomous), according to which V2V resource scheduling and interference management over the PC5 interface are supported based on distributed algorithms implemented between the vehicles.

There are no strict guidelines about the usage of the two modes. However, Mode 3 only applies to *in-coverage* conditions, whereas Mode 4 could work both in *in-coverage* and *out-of-coverage* scenarios. Mode 3 guarantees collision avoidance, thanks to the centralized resource allocation, at the expenses of a larger signaling overhead. On the other hand, Mode 4 requires less signaling but cannot guarantee collision-free transmissions.

Unlike with Mode 4, specifications do not detail a resource management algorithm for Mode 3, and each operator can implement its own solution [25]. A possible option is *dynamic scheduling*, according to which vehicles request subchannels to the eNodeB for each packet transmission. Such an approach incurs signaling overhead, and entails a latency due to the fact that vehicles must wait scheduling grants from the eNodeB before using subchannels. As an alternative, with *semi-persistent scheduling* (SPS) the eNodeB can reserve subchannels for the periodic transmissions of a vehicle. This relieves the vehicle of the signaling burden for periodic resource requests and of the wait for resource grants. However, SPS comes with its own inefficiencies, especially when data patterns (e.g., packet size, packet periodicity) may vary, as discussed in [21], e.g., to accommodate different platoon needs.

## 4. Platooning in Cellular V2X

In this section, we first describe the main reference scenario and assumptions. Then, the main steps of CAM exchange within a platoon are presented, before detailing the addressed scheduling schemes. Finally, we discuss the impact of different network-level resource-allocation settings on the platooning behaviour.

### 4.1. System Model

As a reference scenario for our study, we consider a road where up to *N* platoons are under the coverage of one serving eNodeB. Platoons contain *L* vehicles (*L* being the *platoon size*). Vehicles’ length is *l*, and *d* is the inter-vehicle distance or intra-platoon gap (including the length of one vehicle), which represents the length of the link to be covered through V2V communication. The physical road length occupied by a platoon is (L−1)·d+l (Figure 1). The platoon is assumed to be of fixed size, i.e., joining/leaving procedures are not considered here.

A *predecessor-leader* controller strategy is implemented, which guarantees better stability compared to other strategies. Information exchanged in the platoon is conveyed in CAMs and needs to be periodically updated by the PL (every update cycle of *T* seconds) to satisfy the demands of the controller algorithm in each vehicle. *T* must be kept in the order of 10 ms–100 ms; i.e., the update frequency must be at least 10 Hz. Ensuring shorter update cycles (more frequent CAM updates) allows a platoon to travel faster without incurring the risk of collisions.

We assume that resources are allocated according to Mode 3. Thanks to the eNodeB-assisted resource allocation, the reliability and latency of the platooning application can be more easily controlled. The eNodeB is responsible for allocating subchannels for V2X communications on each TTI. V2X communications occur on dedicated resources. This way, transmissions from non-V2X devices do not interfere with V2X ones. In order to transmit, a VUE (either a PL or a PM) needs to be allocated a number of RBs that depends on the CAM size and the MCS selected for the transmission. We will discuss later how the eNodeB should select the MCS.

### 4.2. CAMs Exchange in the Platoon

CAMs exchange in the platoon occurs cyclically. Each iteration is called an *update cycle*, and is triggered by the PL. Similarly to [24], it includes two steps:

- PL-to-PMs communications. Initially, a PL transmits a CAM to all the PMs.
- PM-to-PM communications. Once the CAM from the PL is received by the platoon, each PM transmits its own CAM to update the one just behind.

At the end of the update cycle, after all vehicles have obtained the data, they compute their next acceleration, based on the measured distance to the respective precedent vehicle (as provided by on-board sensors), and the data received from the preceding vehicle and the PL. Then, all vehicles simultaneously instruct their actuators according to the obtained data.

The first transmission step (PL-to-PMs) is a broadcast transmission. The second one (i.e., the PM-to-PM) entails *several* unicast transmissions. Two different schemes to schedule them can be envisaged.

**Figure 2:**
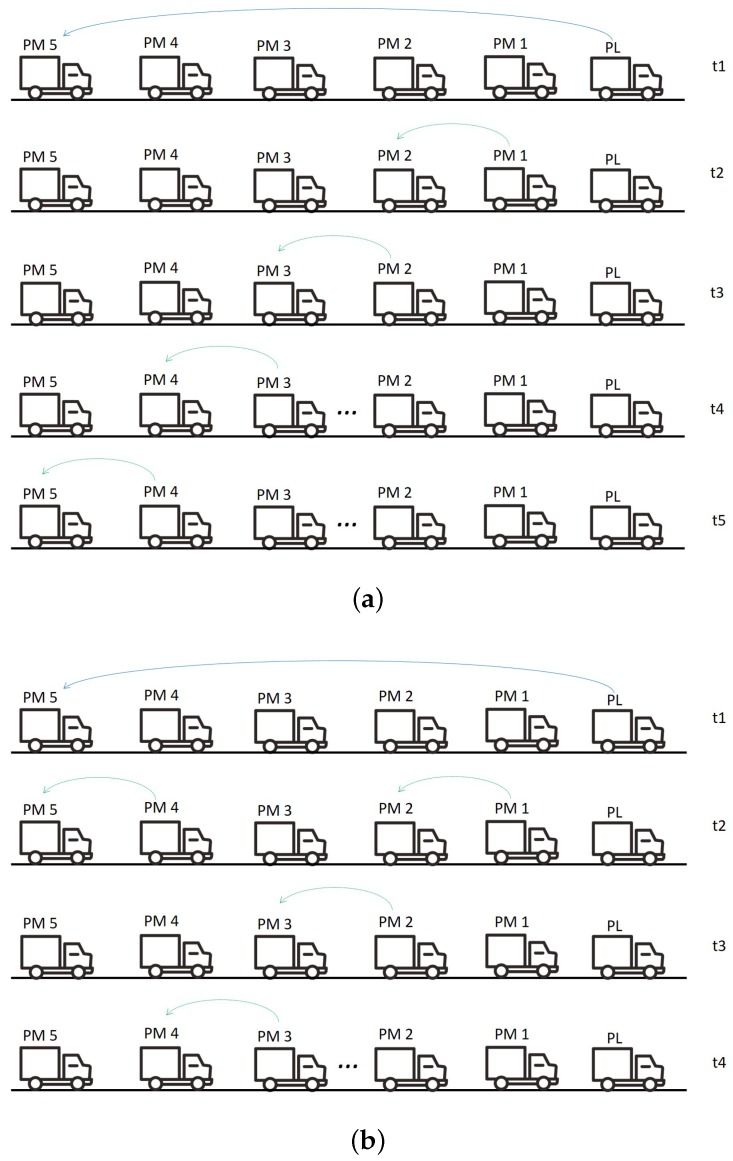
Exemplary CAMs exchange within a platoon at a given update cycle. (**a**) Sequential CAM transmission mode; (**b**) Simultaneous CAM transmission mode.

**Sequential transmission**. Each PM *i* transmits its data *in sequence*, after receiving the CAM from PM i−1. In particular, at update cycle $T_{c}$, PM *i* transmits to the respective follower, PM i+1, the actual acceleration, $a_{i}\left(T_{c}\right)$, as computed at the end of the previous cycle $T_{c}-1$. This scheme resembles the one described in [7] for IEEE 802.11p and is graphically sketched in Figure 2a. Sequential transmissions by adjacent PMs are typically enforced in the literature, when considering TDMA schemes on top of IEEE 802.11p [3,6,8].

**Simultaneous transmission**. Since the information sent by each PM in its CAM at update cycle $T_{c}$ is the acceleration *computed in the previous cycle*, $T_{c}-1$, there is no need to impose a sequence on the transmission of CAMs by PMs within the same update cycle. In other words, the new acceleration computation by each vehicle does not rely on information conveyed in the CAM from the preceding vehicle in the same cycle. A CAM exchange adhering to this scheme is exemplified in Figure 2b, where multiple PMs, sufficiently spaced apart, simultaneously transmit their CAMs using the same RBs under tight control of the eNodeB. Despite the behaviour of the controller algorithm does not demand for sequential transmissions, the viability of the alternative simultaneous approach has not been investigated so far, either for IEEE 802.11p or for C-V2X. This is mainly because the sequential scheme was deemed to better fit the *distributed* IEEE 802.11p protocol. When considering, on the contrary, the *centralized* architecture of C-V2X targeted in our work, we can argue in favour of simultaneous intra-platoon CAM transmissions, as long as they are scheduled to be interference-free by the eNodeB, as clarified in the following.

### 4.3. Mapping to C-V2X

The platoon CAM exchange described above is implemented on top of the C-V2X protocol stack.

First, transmission of a CAM requires that the VUE has some RBs allocated. As mentioned in Section 3, resources can be allocated either dynamically or using SPS, with different performance.

**Figure 3:**
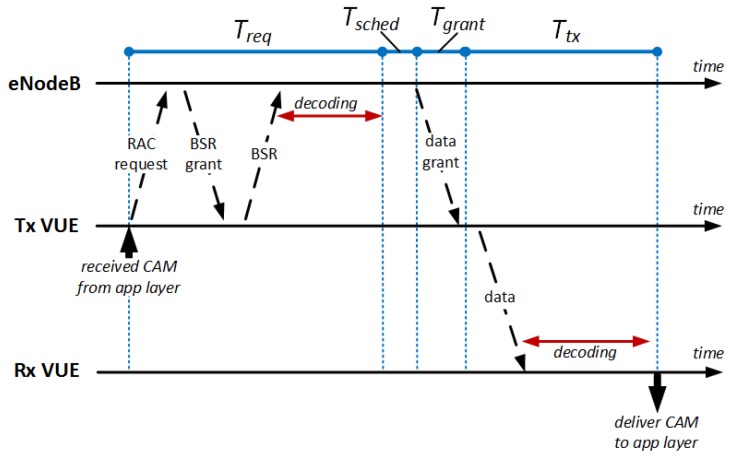
Handshake for obtaining frequency resources.

*Dynamic scheduling* requires non-negligible signaling overhead for each CAM transmission. With dynamic scheduling, a VUE has to explicitly request a transmission grant to the eNodeB for each CAM, using the Random ACcess (RAC) mechanism. The delay between the RAC request by the transmitting VUE and the reception of the actual data transmission by the receiving VUE is unavoidable and lasts 15 ms, in the best case. With reference to Figure 3, the transmitting VUE must send a RAC request to the eNodeB, which in turn grants enough space for it to send its Buffer Status Report (BSR). The BSR reports the amount of bytes that the VUE needs to transmit and it allows the eNodeB to know how many RBs need to be granted to the VUE for data transmission. This handshake lasts $T_{req}$ TTIs. Then, the eNodeB performs the scheduling operations, i.e., it allocates the required number of RBs in $T_{sched}$ subsequent TTIs. $T_{grant}$ is the time required by the eNodeB to issue the transmission grant to the VUE, which indicates which RBs to use for transmitting the CAM. Once the VUE has received the grant, it can transmit the data, which lasts $T_{tx}$. While $T_{grant}$ and $T_{tx}$ can be considered constant, $T_{req}$ and $T_{sched}$ cannot. In fact, collisions during the RAC procedure may occur if different VUEs perform simultaneous RAC requests using the same resources. In this case, the eNodeB grants resources to one of them, whereas the other colliding VUEs, which do not receive a reply from the eNodeB within the expected time window, re-iterate the requests after a backoff. $T_{sched}$, instead, depends on both the BSR and the MCS selected for the transmission, as well as the possible presence of other V2X transmissions.

On the other hand, SPS drastically reduces the signaling overhead for RBs assignment, since VUEs can obtain periodic grants by the eNodeB, based on (periodic or on demand) collected information from the VUEs (about position, channel status, etc.). However, SPS is inflexible, since it is not able to accommodate rapidly the time-varying transmission needs of the VUEs, both from application and resource-allocation perspectives. In fact, the size of CAM and the periodicity of the transmissions can vary whenever the platoon status changes (e.g., when a vehicle joins/leaves the platoon; when changing speed/intra-platoon gap requires modification in the controller algorithm cycle). Likewise, interference or the channel conditions conditions may vary over time, hence the eNodeB should be able to timely change the MCS used by VUEs for transmission.

Another potential advantage of using dynamic scheduling instead of SPS is that it allows the network to better exploit *spatial frequency reuse*. Frequency reuse schemes (like the one in [31]) allow two or more D2D (V2V in our study) transmissions to be scheduled on the same RBs, if their mutual interference is weak. This allows the eNodeB to save precious frequency resources that could be re-utilized for serving other V2X communications and reducing the delay before transmission. With dynamic scheduling, the eNodeB can better control the interference level by selecting, at every TTI, which VUEs can use the same resources, i.e., by avoiding to share RBs among highly-interfering VUEs. In this way, *inter-platoon frequency reuse* can be exploited if two platoons are far from each other, and also frequency reuse can be enforced *among VUEs of the same platoon* if their transmit power is sufficiently low and/or transmitting PMs are far enough. Intra-platoon frequency reuse is not applicable if PMs’ transmissions are sequential, in which case they occur at different time instants. Note that frequency reuse could be enabled for SPS mode, too. However, given the high-mobility characteristics of V2V scenarios, it is difficult for the eNodeB to predict how much interference two VUEs will suffer for the entire duration of the SPS grant.

For the above reasons, we only consider dynamic scheduling as allocation scheme in the following and leave the investigation of SPS (e.g., studying the impact of the SPS grant duration) as a future work.

As mentioned in the previous subsection, CAM exchange foresees two steps, i.e., PL-to-PMs and PM-to-PM. These two logical connections have to be mapped to C-V2X communication modes. According to the semantics of the two steps, we employ point-to-multipoint (P2MP) V2V communications for the PL-to-PM step, whereas we select point-to-point (P2P) V2V communications for the PM-to-PM phase. We now discuss the issues related to the two modes separately.

#### 4.3.1. PL-to-PMs Step

This phase is realized through P2MP V2V communications. This way, the PL only needs to send its CAM *once* and all the interested receivers can decode it.

The first problem is to determine the set of destinations, i.e., vehicles interested in receiving and decoding the message. In fact, PMs belonging to different platoons can be in the transmission range of the transmitting PL. It is not desirable that these PMs receive the CAM, since it poses additional workload on these devices and, in the worst case, might affect the controller algorithm (e.g., changing their speed to a wrong value). This problem can be addressed at the application layer. To this aim, a Platoon ID field is added to the CAM messages. Platoon ID is a unique string that is used to differentiate between different platoons. Similar to previous studies [32], we set Platoon ID to be vehicle ID of the PL. When a vehicle joins a platoon, besides other information, it acquires from the PL the Platoon ID reserved for that platoon, in order to receive CAMs from the PL of that platoon. When a PL needs to send its CAM to all its PMs, it only specifies the Platoon ID in the transmitted CAM. All the PMs belonging to the platoon receive the CAM, whereas other vehicles discard it. A possible improvement could be reached by enlisting the aid of the network infrastructure: if a cell allocates a multicast MAC ID to each platoon traversing it, CAM messages of a platoon can be encapsulated into MAC-level transmissions having that MAC ID as a destination address. This way, other platoons would discard the CAM messages directly at the MAC layer, without having to go all the way up through the stack. Moreover, since cellular transmissions are cyphered, this solution could be used to enhance the security.

Another issue is the reliable delivery of the PL’s CAM to all the PMs of the platoon. In fact, it is desirable that all the PMs belonging to the platoon receive the CAM with at least a certain probability. In ideal conditions (i.e., with no interference), the reception range depends on the CAM size, the PL’s transmit power and the selected MCS. Given these parameters, it is possible to find the maximum platoon size *L* that can be covered with a single transmission from the PL. If the reception range does not cover the entire platoon, one of the PM needs to relay the CAM to other PMs. This implies some mechanism that allows one PM to be elected as a relay (additional message exchange), or a dissemination mechanism like the one in [33]. Considering that the CAM size depends on the amount of information that needs to be sent and that transmit power is usually fixed by the standard or implementation constraints, the only way to adapt the reception range is to manipulate the MCS. The latter is not a trivial task, since there is a tradeoff between the reception range and radio resources consumption. Using lower MCSs allows the PL to reach farther PMs, but it comes at the cost of more RBs per transmission. In fact, we recall that the number of bits that can be sent in one RB decreases with the MCS. On the other hand, higher MCSs allow the eNodeB to reduce the number of RBs required for a transmission, but it will also decrease the reception range.

Finally, P2MP V2V transmissions are not acknowledged at the MAC level, i.e., there is no Hybrid Automatic Repeat Request (H-ARQ) to recover corrupted transmissions. This means that application-level retransmissions should be foreseen in order to improve the correct reception probability at the PM side. Clearly, adding retransmissions might increase the duration of the CAM cycle.

#### 4.3.2. PM-to-PM Step

This step requires that one PM sends its CAM to the subsequent PM only, hence P2P V2V is the most suitable communication mode. Like P2MP V2V communications, also P2P ones require that the MCS is selected by the eNodeB. Regarding the reception range, the same considerations done for P2MP V2V communications are valid. Link adaptation for P2P links can be performed dynamically be the eNodeB, which can take advantage of CQI periodically reported by the PMs. This allows the eNodeB to allocate RBs using the MCS that suits better the considered V2V link. However, the accuracy of the MCS selection depends on the frequency of CQI reporting: even if the distance between PMs belonging to the same platoon is almost constant, the variations of the surrounding environment in such high-mobility scenario may cause the interference to vary even within the same subframe. On the other hand, frequent reporting (e.g., every millisecond) is hardly feasible due to the large signaling overhead.

When a vehicle joins a platoon, in addition to the Platoon ID, it also receives information about the relative position within the platoon, i.e., the PM ID. PMs are identified through a sequential number. When a PM, *i*, needs to send its CAM to its follower, i+1, it specifies the Platoon ID and the PM identifier of its follower in the transmitted CAM. Only PM i+1 belonging to the platoon Platoon ID will receive the CAM, whereas other PMs discard it.

As far as transmission timing is concerned, the MAC layer of the PM has to initiate the handshake procedure with the eNodeB in order to obtain frequency resources. As mentioned before, the time between the resource request and the reception of the CAM is not lower than 15 ms. This means that up-to-date information can be propagated in the same cycle only if a delay of 15 ms per hop is tolerated. This is not the case for the proposed simultaneous transmission scheme, according to which all PMs can start the handshake procedure with the eNodeB soon after receiving the CAM from the PL. It will be then the eNodeB to decide which PMs could transmit, over which resources, *even at the same time*, possibly according to frequency reuse constraints. Moreover, P2P V2V communications can exploit a configurable number of H-ARQ retransmissions to improve reliability. This is possible if the receiving endpoint of the V2V link sends a MAC-level acknowledgement in response to a transmission. The way this can be achieved is outside the scope of this work, although we refer the interested reader to [34], which discusses H-ARQ-related issues in D2D communications. Anyway, a retransmission is scheduled by the eNodeB in case of a negative acknowledgement, eight TTI after the previous transmission. Obviously, H-ARQ retransmissions affect the time needed to complete a CAM cycle. Moreover, taking into account H-ARQ retransmissions at the application layer is not trivial if sequential transmissions are used.

## 5. Theoretical Bounds

This Section provides a numerical comparison between the sequential and simultaneous transmission schemes in terms of the time required to complete a CAM cycle when varying the composition of the platoons, CAM size and network parameters. The analysis is intended to provide theoretical bounds for such metrics, while neglecting the specific channel dynamics and spatial dimension, affecting interference and propagation conditions, i.e., all platoons are assumed to be served by the same eNodeB and all platoon vehicles are under each other coverage.

**Sequential transmission.** The selection of the CAM cycle duration must take into account that the PM-to-PM step in a platoon with L−1 PMs is $T_{PM-to-PM}^{seq}=\left(L-1\right)$·Δ in the best case, where $\Delta =T_{req}+T_{sched}+T_{grant}+T_{tx}$ is the time required to perform one CAM transmission considering the handshake between the PM and the eNodeB for obtaining the frequency resources, as shown in Figure 3. Let us consider $T_{req}$, $T_{grant}$ and $T_{tx}$ as constant values. In this case, Δ is minimized if $T_{sched}=1$ TTI for each PM *i* belonging to the platoon, i.e., there are enough RBs to schedule the transmission of PM *i* in one TTI. This can be achieved depending on the total number of PMs (considering other platoons, too) that are simultaneously active; this is because we assume dedicated resources for V2X platooning communications. Assuming that one TTI has *M* available RBs and that the transmission of one CAM needs D<M RBs, the maximum number of PMs that can transmit in the same TTI is $K_{max}<\left\lceil M/D\right\rceil$. This means that Δ is minimized if there are $K\leq K_{max}$ synchronized platoons. Otherwise, the eNodeB cannot schedule all the PMs in one TTI and some transmission grants must be allocated during subsequent TTI(s), hence increasing $T_{sched}$. The overall CAM completion time is computed by adding a Δ contribution to $T_{PM-to-PM}^{seq}$ to account for the PL CAM transmission.

**Figure 4:**
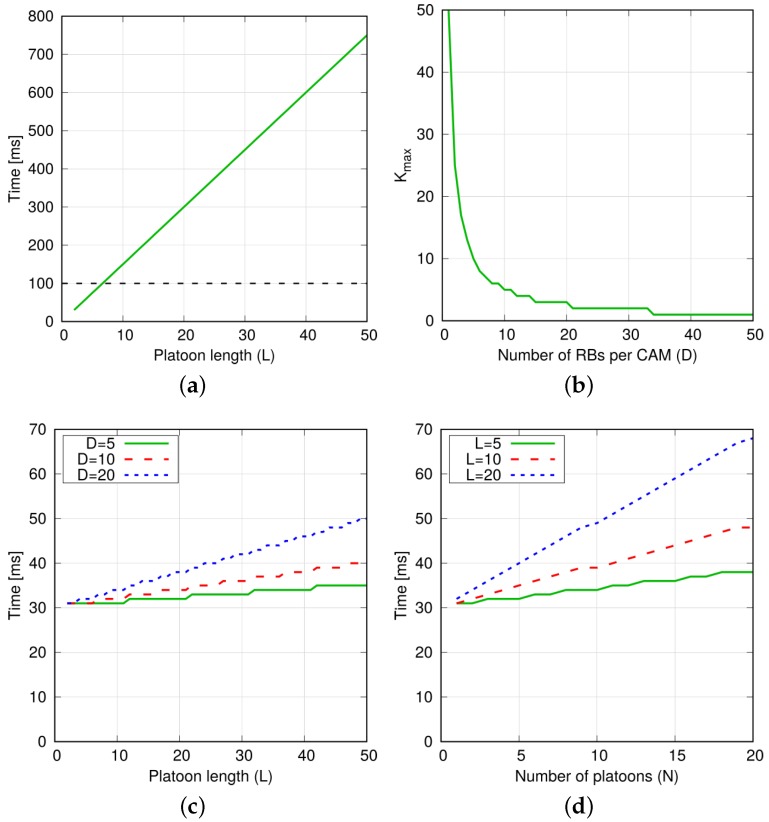
Theoretical bounds for the sequential and simultaneous transmission schemes (M = 50). (**a**) CAM completion cycle in case of sequential transmission (*N* = 1); (**b**) Maximum number of synchronized platoons; (**c**) CAM completion cycle in case of simultaneous transmission (*N* = 1); (**d**) CAM completion cycle in case of simultaneous transmission (D = 5).

Results in Figure 4a show that only platoons with few vehicles (around 7) can be supported while ensuring that the CAM completion time is below 100 ms. The maximum number of synchronized platoons $K_{max}$ as a function of *D* is reported in Figure 4b. In order to provide exemplary values for *D*, let us consider a 300-byte CAM and a CQI equal to 7. According to tables in [17] and after some straightforward computations, we obtain that the eNodeB has to allocate 14 RBs, hence D=14. Instead, if we consider perfect channel quality, i.e., CQI=15, we obtain D=4. Under such range of *D* settings, $K_{max}$ is in-between 5 and 10.

**Simultaneous transmission.** In such a case, all the PMs of a platoon start the CAM transmission at the same time. Assuming that $T_{req}$, $T_{grant}$ and $T_{tx}$ stay constant for all PMs, the duration of the PM-to-PM phase for one platoon depends on $T_{sched}$, i.e., the time required to complete the scheduling for all the PMs, which in turn depends on both their number and the CAM size. In more details, the number of RBs required to schedule the whole platoon is (L−1)·D. Since the eNodeB can allocate at most *M* RBs in one TTI, $T_{sched}=\left\lceil \frac{(L-1)\cdot D}{M}\right\rceil$. As a result, we obtain $T_{PM-to-PM}^{sim}=T_{req}+\left\lceil \frac{(L-1)\cdot D}{M}\right\rceil +T_{grant}+T_{tx}$. Again, this is the ideal case in which the eNodeB is serving one platoon only. In the worst case, *N* platoons are synchronized, i.e., their PMs transmit at the same instant. In this case, the time required to accommodate all transmissions becomes $T_{PM-to-PM}^{sim}=T_{req}+\left\lceil \frac{N\cdot (L-1)\cdot D}{M}\right\rceil +T_{grant}+T_{tx}$. In general, if the platoons have different length $L_{j}$, then $T_{PM-to-PM}^{sim}=T_{req}+\left\lceil \frac{\sum_{j=1}^{N}\left(L_{j}-1\right)\cdot D}{M}\right\rceil +T_{grant}+T_{tx}$. As in the previous case, the overall CAM completion time is computed by adding a Δ contribution to $T_{PM-to-PM}^{sim}$.

Figure 4c reports the CAM completion cycle when varying *D* and *L*. Values in the range 30–40 ms are achieved under for D≤10 and up to 50 vehicles per platoon (overestimated platoon length), which are significantly lower than the ones achieved in the sequential transmission case. Figure 4d shows that all CAM transmissions are accommodated in less than 50 ms when there are 10 platoons, each one with 20 vehicles.

## 6. Performance Evaluation

### 6.1. Simulation Tool

Simulations are carried out using SimuLTE [26], a system-level simulator based on OMNeT++ [27] that models the full LTE protocol stack and includes support for P2P and P2MP V2V communications, while accurately capturing channel dynamics. In order to assess the performance of the proposal described in Section 4.2, we extended SimuLTE so as to support CAM exchange within a platoon and the latest C-V2X specification when considering the proposed scheduling modes for CAM transmissions. In particular, modifications affect the following modules.

**Platoon CAM application**. We implemented two application modules to mymic the CAM exchange. The former models the behavior of the PL and sends one CAM to its PMs at the beginning of every CAM cycle, using P2MP V2V transmissions. In turn, the application running at the PMs receives the PL’s CAM and sends one CAM to the following PM using a P2P V2V transmission. We assume that each platoon is formed at the beginning of the simulations and that PMs know the address of the following vehicle, hence we do not simulate the platoon’s joining phase. If a PM does not receive the CAM from the PL (e.g., due to unsuccessful decoding), it is configured to send its CAM in any case (i.e., with the latest speed/acceleration information available) at the scheduled time. In other words, the PM does not wait for receiving the CAM from the PL before transmitting, otherwise it could be stalled in case of packet loss. This is practical because PMs are aware of the timing of the update cycle, that is provided by the PL during the joining phase.

**Propagation model**. The propagation model in [35] has been implemented to compute the path loss, with parameters set to match a highway environment and a convoy path loss. The same model is also considered as propagation scenario #PS9 of the METIS (Mobile and Wireless Communications Enablers for the 2020 Information Society (https://www.metis2020.com/) project. In particular, the path loss for a receiver at a distance *x* from the intended transmitter is computed as follows:

(1) $$PL\left(x\right)=PL_{0}+10\cdot n\cdot log_{10}\frac{x}{x_{R}}+Y_{\sigma }$$

where $PL_{0}$ is the reference path loss equal to 63.3 dB, *n* is the path loss exponent set to 1.77, $x_{R}$ is the reference distance (10 m), Yσ is a normally distributed random variable with mean μ equal to 0 and standard deviation σ equal to 3.1.

### 6.2. Scenario Configuration

We consider a 2000 m-stretch of a highway, composed of four lanes and traversed by platoons only. Cellular connectivity is provided by one eNodeB, whose V2X-reserved bandwidth is 10 MHz.

Unless otherwise stated, we simulate *N* PLs and 10×N PMs. The size of each of the *N* platoons is randomly chosen with the constraint that the overall number of vehicles is constant. *N* is varied over the simulations.

Intra-platoon gap is set to d=10 m. CAM cycle duration is T=100 ms and each CAM is 300 bytes at the application layer (hence, excluding extra headers introduced by lower layers). This size matches the needs to accommodate data conveying cooperative manoeuvres and cooperative perception data, according to [22]. As mentioned before, VUEs request resources according to Mode 3, whereas the eNodeB schedules per-TTI resources according to the Max C/I policy. H-ARQ retransmissions are disabled, in order to better understand the interference dynamics and how they are addressed by the eNodeB with proper CQI/MCS settings. For each configuration, we performed ten repetitions of 20 s. The main simulation settings are summarized in Table 1.

### 6.3. Metrics

Our aim is to assess the performance of the proposal, showing that C-V2X communications match the strict latency and reliability requirements of platooning, and evaluating their efficiency in terms of resource usage. In order to do this, we refer to the following set of metrics:

- Probability of successful reception of the PL-to-PM CAM against the distance from the PL. It is derived as the number of successfully received CAMs over the number of transmitted CAMs by the PL.
- Allocated RBs per TTI. It represents the number of RBs per TTI which are allocated to accommodate the transmission of CAMs of all platoon vehicles.
- CAM cycle completion time. It refers to the time required to accommodate all the CAM transmissions (PL to PMs and PM to PM) within a platoon at a given update cycle.
- Ratio of successfully completed CAM cycles. It is computed as the ratio between the number of CAM cycles for which *all* CAMs have been correctly delivered and the total number of CAM cycles.

### 6.4. Results

In the following, results are reported which are achieved under different settings and traffic load (in terms of number of platoons and platoon size). They are presented in an incremental manner, gradually moving towards smarter configurations which make the best of the capabilities of the centralized cellular radio resource orchestration enabled by the usage of Mode 3.

#### 6.4.1. PL-to-PM phase

The first set of results is aimed to shed light on the performance of PL-to-PM communications and on possible settings for the relevant parameters. First, we evaluate the reception range of P2MP V2V communications with different settings of the MCS. To this aim, we consider a scenario with one platoon.

**Figure 5:**
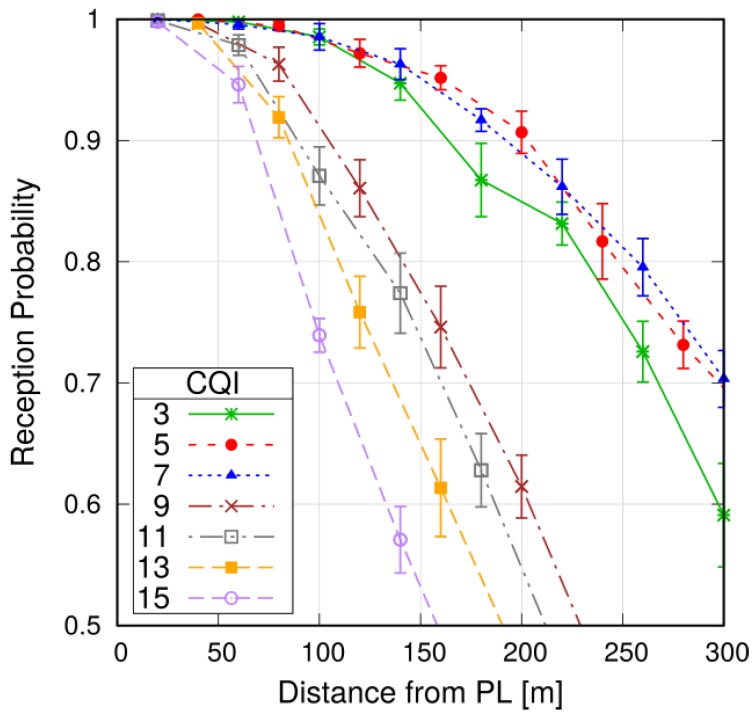
Probability of successful reception of the PL-to-PM CAM, as a function of the distance from the PL (N=1).

Figure 5 reports the probability of successful reception of the PL-to-PM CAM over the distance from the PL for different CQI settings. The metrics decreases with the distance, due to the weaker received signal. Moreover, lower probabilities are observed for higher CQI settings, which are less robust. In particular, at 200 m from the PL, which would resemble the position of a PM at the tail of the platoon (e.g., in case of a platoon with 20 vehicles and intra-platoon gap equal to 10 m), the reception probability is in the order of 0.9, for CQI values equal to or below 7. At a distance of 100 m, for CQI equals to 3, 5, 7, the metrics is very close to 1, thus ensuring very high reliability in the reception of the CAM from the PL. A distance of 100 m from the PL could be the case of a platoon with 10 m-spaced vehicles, which is common platoon setting.

Note that for CQI = 3, reliability is lower than that with CQI = 5–7. This can be ascribed to the fact that, in order to successfully receive a transmission, the receiver needs to correctly decode *all* the RBs used for that transmission (i.e., the error probability is also proportional to the number of used RBs). Since a CAM sent with CQI = 3 occupies many more RBs than with CQI = 5–7 (as shown below), the probability of erroneous reception is larger.

**Figure 6:**
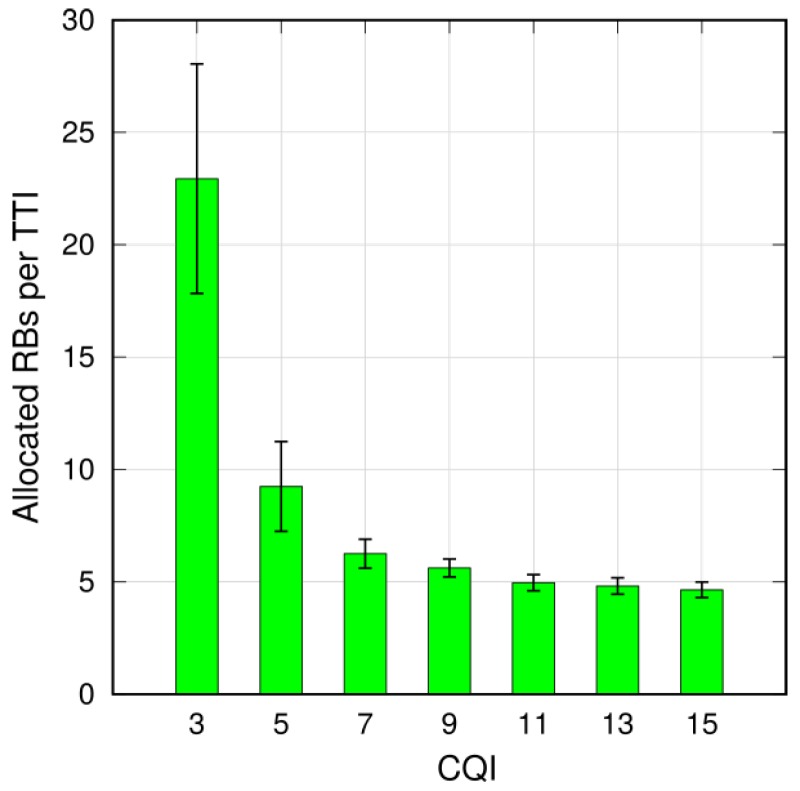
Average allocated RBs per TTI as a function of CQI used by the PLs (N=15).

The choice of the employed MCS significantly affects the number of allocated RBs. For example, when considering a scenario with N=15 platoons, the average number of allocated RBs per TTI is reported in Figure 6. As expected, it is larger for lower CQIs, which ensure a higher robustness at the expenses of a higher transmission overhead. A CQI value equal to 7 achieves a good trade-off in terms of allocated resources and ensured reliable coverage. Henceforth, we will refer to CQI = 7 for the PL-to-PM CAM transmission.

#### 6.4.2. PM-to-PM Phase

We now focus on the PM-to-PM phase in scenarios with multiple platoons, and assess the performance of different configurations and parameters settings.

**Figure 7:**
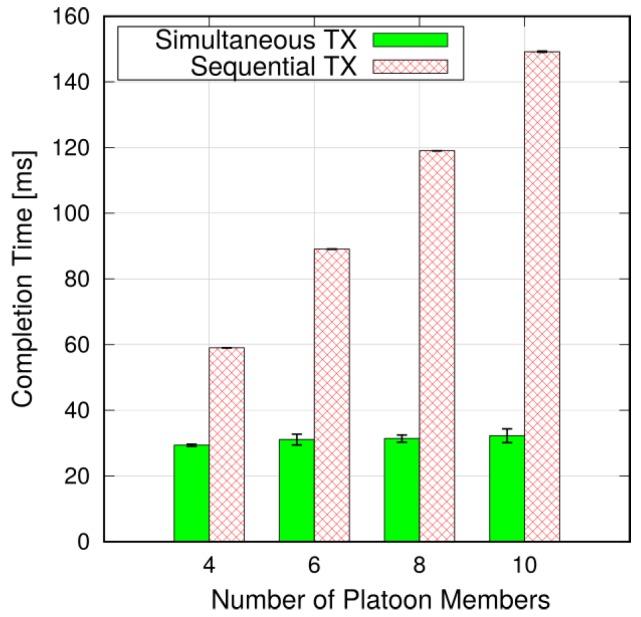
Average completion time of one CAM cycle (N=12).

**Sequential vs. simultaneous transmissions**. We first compare sequential and simultaneous transmission schemes in terms of the average time required to complete a CAM cycle. Considering a scenario with N=12 platoons, Figure 7 shows the average time required to complete a CAM cycle using the two modes. Since the platoon size affects the cycle duration, we group the platoons by the number of PMs and report a different measurement on the x-axis for each group.

As expected, the completion time with sequential transmissions increases linearly with the platoon size. This is obvious, since each PM sends its CAM only after it has received the information from the preceding vehicle. In particular, the time required to obtain the frequency resources (i.e., sending RAC request and reporting the BSR) has to be paid at each hop, hence extending the time to reach the last member of the platoon. When considering platoons with more than six members, the completion time is longer than T=100 ms. On the other hand, the completion time is almost insensitive to the platoon size in case of simultaneous transmissions and it stays at about 30 ms. Such results are in line with the theoretical bounds reported in Section 5. This is because the latency incurred to obtain frequency resources is paid off almost simultaneously for each PM.

**Figure 8:**
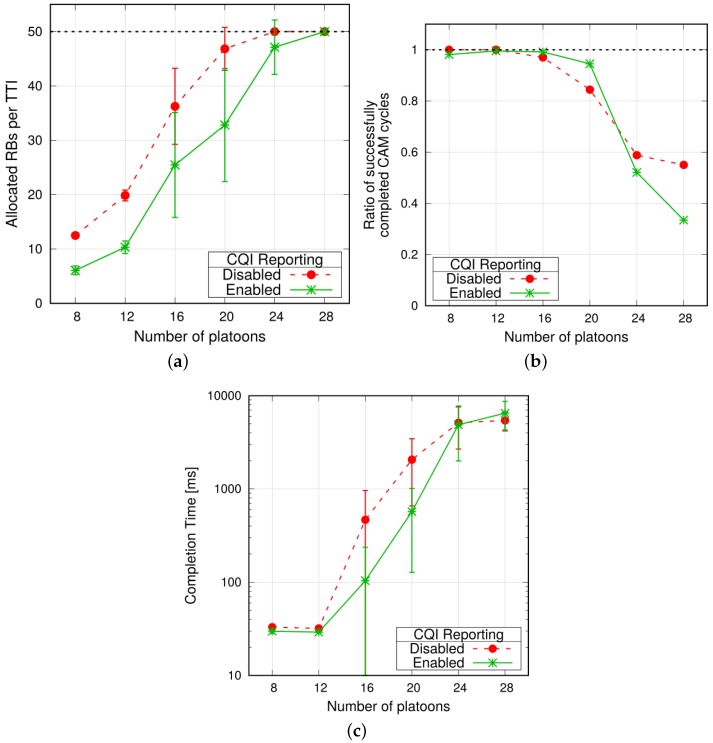
Metrics evaluated with and without CQI reporting, simultaneous transmissions (L=10). (**a**) Average allocated RBs per TTI; (**b**) Ratio of successfully completed CAM cycles; (**c**) Average completion time of one CAM cycle.

**Impact of CQI reporting**. We now consider simultaneous PM-to-PM transmissions and evaluate how the MCS selection for P2P V2V communications affects the performance. The proposal has been evaluated under two different settings: (i) the MCS to be used is decided a priori (curves labeled as *CQI Reporting Disabled* in Figure 8); and (ii) the MCS is set according to the CQI reports from the VUEs (curves labeled as *CQI Reporting Enabled* in Figure 8). In the former case, the eNodeB schedules PM-to-PM transmissions with the same modulation as PL-to-PM ones, which we assumed to be the one corresponding to CQI = 7. In the latter case, each PM periodically reports the *wideband* CQI measured on the P2P V2V link to the eNodeB, i.e., they report a CQI value that corresponds to the average channel quality perceived over *all* the RBs. The reporting period is set to 6 ms (the tuning of the reporting period is outside the scope of the present work).

Given the proximity of the endpoints in a PM-to-PM communication, the CQI reported by the PMs will generally be higher than the one used when reporting is disabled. This results in fewer RBs to be allocated every TTI for a single PM-to-PM transmission. Figure 8a shows the average number of RBs allocated by the eNodeB in a TTI with increasing network load, where we can observe that enabling CQI reporting allows the eNodeB to save RBs. With reporting disabled, the eNodeB starts to approach the saturation at 20 platoons, whereas using CQI reporting the subframe capacity saturates when there are 28 platoons under the eNodeB’s control. When the maximum capacity is approached, the network is unable to serve rapidly all VUEs, hence some update cycles experience large delays or they even fail to complete at all, due to shortage of radio resources. Figure 8b,c show, respectively, the ratio of successfully completed cycles and their corresponding completion time. In order to perform a fair measurement, we consider only platoons with the same length L=10. We observe that before going into a congestion situation, enabling CQI reporting guarantees higher reliability and significantly lower latency.

We also note that with CQI reporting enabled, confidence intervals are quite large at intermediate traffic loads. We ascribe this behavior to different effects of wideband CQI reporting in different simulations runs. In some repetitions, multiple PMs (belonging to either the same or different platoons) transmit at the same time, hence the number of allocated RBs in one TTI is large. This causes the PMs to report smaller values of wideband CQIs, which in turn imply even more allocated RBs. Thus, there is a positive feedback that rapidly brings the network in saturation. In other runs, transmissions from PMs are more separated in time and the utilization of per-TTI bandwidth stays smaller. In this case, the positive feedback described above is not triggered and the network does not saturate.

**Impact of frequency reuse**. Finally, we assess the benefit of reusing frequency resources among PM-to-PM transmissions. As anticipated in Section 4.2, a feature envisaged for V2V (and more generally D2D) communications is to allow two or more transmissions to share the same bandwidth if their mutual interference is low. To this aim, we use the allocation scheme proposed in [31], where we assume that the eNodeB is able to build a graph, whose vertices represent the communication links between PMs and an edge exists between vertices if the mutual interference when they use the same RBs is above a configurable threshold. In the following, we refer to the latter as *conflict threshold*. When allocating resources, the eNodeB avoids to grant the same RBs to PM-to-PM communications that are connected by an edge in the graph. Since the tuning of the conflict threshold affects the construction of the graph (hence, the scheduling), we compare the scheme with no reuse against two scenarios where the conflict threshold is set to −75 and −90 dBm, respectively. In these simulations, CQI reporting by the PMs to the eNodeB is enabled, which is shown to be efficient and effective, before reaching saturation of resources.

**Figure 9:**
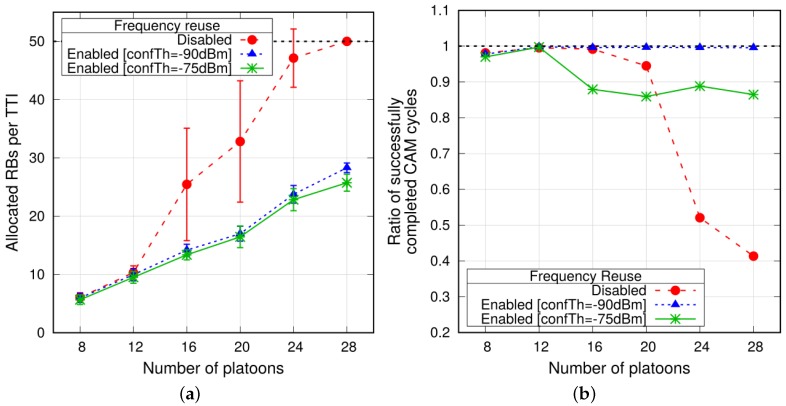

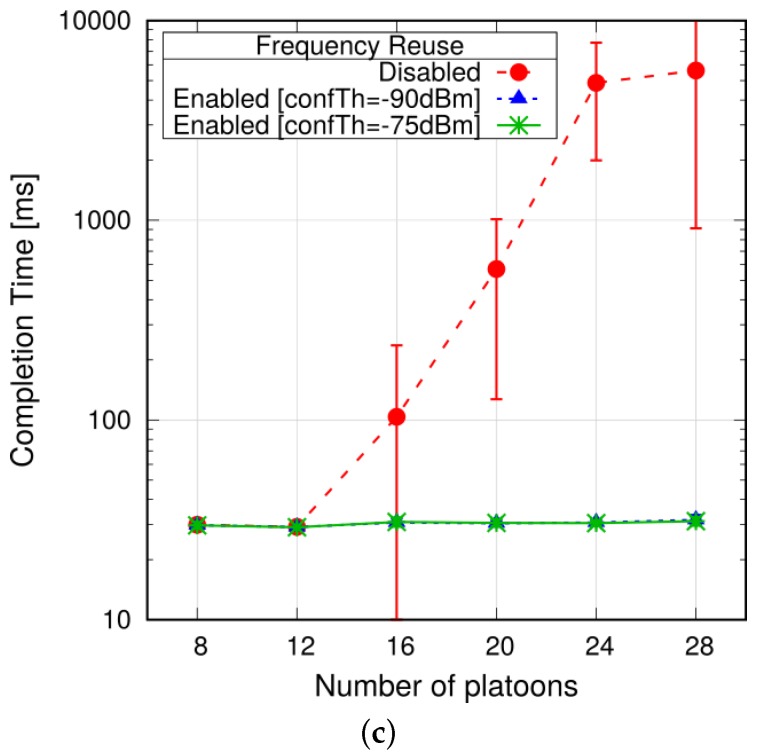
Metrics evaluated with and without frequency reuse, simultaneous transmissions and CQI reporting enabled (L=10). (**a**) Average allocated RBs per TTI; (**b**) Ratio of successfully completed CAM cycles; (**c**) Average completion time of one CAM cycle.

The average allocated RBs per TTI are reported in Figure 9a. As expected, frequency reuse allows the eNodeB to save resources, which can be exploited to serve a larger number of VUEs. In fact, the eNodeB is now able to support also 28 platoons using 60% of the bandwidth on average. Note that the larger conflict threshold gives a reduction of the number of allocated RBs, which becomes more remarkable as the number of platoons increases, at the cost of slightly lower reliability, as shown in Figure 9b, which reports the ratio of successfully completed cycles for platoons composed of nine PMs. The benefits of frequency reuse are also evident in terms of the average completion time, which stays well below the considered CAM cycle period *T* (Figure 9c), even when considering a higher number of platoons.

## 7. Discussions

The conducted analysis helps us to derive some key insights into the potential of the C-V2X technology in supporting platooning applications.

Results clearly shows that enabling the simultaneous PM-to-PM transmission mode, after the PL transmits its CAM, significantly reduces the CAM cycle completion time. Such a feature uniquely characterizes the conceived proposal, which oversteps the sequential transmission mode reminiscent of TDMA solutions built on top of IEEE 802.11p.

In addition, when moving to the specific C-V2X settings, the main findings are as follows. CQI reporting is shown to highly improve the resource utilization. Adapting the MCS to the actually experienced channel quality of the receiver, makes more efficient the short-distance PM-to-PM transmissions, overall reducing the interference.

In addition, enabling frequency reuse, which is the among the key features of V2V-enabled cellular networks compared to the infrastructureless 802.11 technology, ensures high-reliability, low latency and high capacity. Thus, it is particularly promising in the view of enabling platoon vehicles to exchange CAMs very frequently thus, able to keep very small inter-vehicle distances.

In particular, with values of the completion of a CAM cycle in the order of 30 ms, the transmission of 30 CAMs/second could be accommodated as recommended in [22].

The achieved encouraging results coupled with the recent and planned advancements of the cellular technology in the roadmap towards 5G new radio make us confident that there is room for improvements.

The latency incurred by the dynamic scheduling approach could be further reduced if a different numerology is considered, i.e., TTI values shorter than 1 ms. Such an option is under investigation in 3GPP Release 15 and its benefits have been preliminarily studied in [36,37]. Thus, in the near future the usage of the dynamic scheduling could be incentivized. Additionally, lighter signaling procedures could be devised aimed to reduce the latency incurred in getting the resource grants from the eNodeB prior to CAM transmissions. The comparison of such techniques against the semi-persistent scheduling scheme would be a subject matter of future works.

With such advancements, up to 50 and 100 CAMs per second could be further supported in the view of high-density platooning scenarios with high level of vehicle automation [22].

Another issue that needs to be addressed for platooning exploiting C-V2X communications is the management of mobility. In fact, a platoon moving on a highway will traverse multiple cell areas and it is likely that vehicles are under control of different eNodeBs. In this case, continuity of inter-cell V2V communications should be guaranteed, for instance providing coordination mechanisms among neighboring eNodeBs. On the other hand, *bulk handover* solutions could be envisaged to allow all vehicles in a platoon to perform handover at the same time, reducing both latency and the amount of signaling.

## 8. Conclusions

In this work we investigated the potential of the recently specified C-V2X technology to match the latency and reliability demands of platooning application. In particular, we consider two ways of implementing dynamic scheduling for CAM transmissions within platoons, currently unspecified by 3GPP: the sequential mode, that is somehow reminiscent of TDMA solutions based on IEEE 802.11p, and the simultaneous mode with spatial frequency reuse enabled by the tight control of the eNodeB. The latter reduces the latency in the update cycle in each platoon vehicles controller fed by the exchanged CAMs. After a preliminary evaluation through theoretical bounds, a wide set of results achieved through a system-level simulator are reported under different configurations and load settings, with the ultimate goal to showcase the benefits of the C-V2X network-orchestrated radio resource allocation mode and our proposed enhancements specifically targeting platooning applications. Interesting findings are provided and hints about future works shortly discussed.

## Figures and Tables

**Table 1:** Main settings.

Layer	Parameter	Value
Platoon and road settings	Number of lanes	4
	Road length	2 Km
	Number of platoons (*N*)	varying
	Platoon size (*L*)	random
	Intra-platoon gap (*d*)	10 m
	CAM size	300 Bytes
	CAM update cycle (*T*)	100 ms
Network settings	Available bandwidth	10 MHz (50 RBs)
	VUEs’ tx power	10 dBm
	Resource allocation mode	Mode 3
	Scheduling policy	Max C/I
